# Genetic Differentiation Revealed by Selective Loci of Drought-Responding EST-SSRs between Upland and Lowland Rice in China

**DOI:** 10.1371/journal.pone.0106352

**Published:** 2014-10-06

**Authors:** Hui Xia, Xiaoguo Zheng, Liang Chen, Huan Gao, Hua Yang, Ping Long, Jun Rong, Baorong Lu, Jiajia Li, Lijun Luo

**Affiliations:** 1 Shanghai Agrobiological Gene Center, Shanghai, China; 2 Center for Watershed Ecology, Institute of Life Science and Key Laboratory of Poyang Lake Environment and Resource Utilization, Ministry of Education, Nanchang University, Nanchang, China; 3 Ministry of Education Key Laboratory for Biodiversity and Ecological Engineering, Fudan University, Shanghai, China; Department of Agriculture and Food Western Australia, Australia

## Abstract

Upland and lowland rice (*Oryza sativa* L.) represent two of the most important rice ecotypes adapted to ago-ecosystems with contrasting soil-water conditions. Upland rice, domesticated in the water-limited environment, contains valuable drought-resistant characters that can be used in water-saving breeding. Knowledge about the divergence between upland and lowland rice will provide valuable cues for the evolution of drought-resistance in rice. Genetic differentiation between upland and lowland rice was explored by 47 Simple Sequence Repeats (SSRs) located in drought responding expressed sequence tags (ESTs) among 377 rice landraces. The morphological traits of drought-resistance were evaluated in the field experiments. Different outlier loci were detected in the *japonica* and *indica* subspecies, respectively. Considerable genetic differentiation between upland and lowland rice on these outlier loci was estimated in *japonica* (*Fst* = 0.258) and *indica* (*Fst* = 0.127). Furthermore, populations of the upland and lowland ecotypes were clustered separately on these outlier loci. A significant correlation between genetic distance matrices and the dissimilarity matrices of drought-resistant traits was determined, indicating a certain relationship between the upland-lowland rice differentiation and the drought-resistance. Divergent selections occur between upland and lowland rice on the drought-resistance as the *Qsts* of some drought-resistant traits are significantly higher than the neutral *Fst*. In addition, the upland- and lowland-preferable alleles responded differently among ecotypes or allelic types under osmotic stress. This shows the evolutionary signature of drought resistance at the gene expression level. The findings of this study can strengthen our understanding of the evolution of drought-resistance in rice with significant implications in the improvement of rice drought-resistance.

## Introduction

How plants have developed resistance to biotic and abiotic stresses is a fundamental question in plant biology. To address this question, plant ecotypes grown in contrasting environments are studied as they are likely under divergent selections, resulting in adaptive divergence [1]. Although ecotypes of wild species adapted to different ecosystems are served as ideal systems for studying the evolution of resistance to biotic and abiotic stresses [2]–[5], literatures on different ecotypes of a crop to a specific stress are still rare.

The Asian cultivated rice (*Oryza sativa* L.) is one of the most important cereal crops in the world by providing stable food for >50% of the total global population (ref). However, rice production has encountered severe challenges due to frequent droughts and water shortages [6]. The utilization of natural variations of water-saving and drought-resistant characters in upland rice is an effective solution to improve the drought-resistance for rice [7]–[8]. Upland rice has adapted to the water-limited and rain-fed rice ecosystems, facing the higher risk of drought [9]. On the contrary, lowland rice is commonly planted in fields with irrigation facilities with relatively lower risks of drought. Upland rice may accumulate more drought-resistant genetic variances than lowland rice, leading to potential adaptive divergence of this rice ecotype on drought-resistance [7]. Therefore, studying differentiation between upland and lowland rice provides ideal systems for understanding the evolution of drought resistance and exploring drought-resistant genetic resources in crops. Previous attempts using neutral markers to explore differentiation between upland and lowland rice were not very successful [10]–[11], while another study using the functional-based markers has detected some ecotype-specific genetic features between upland and lowland rice [12].

The simple sequence repeats (SSRs) located in the ESTs (expressed sequence tags) are developed and widely applied in population genetics and breeding since the last decade [13]. The expression of drought-responding EST-SSRs is up- or down- regulated by drought stress. Although these genic SSRs are not always selective among ecotypes, they have a higher probability of being associated with the drought-resistance. In this study, 47 drought-responding EST-SSR loci were selected to explore the genetic differentiation between upland and lowland rice ecotypes from China. The questions to be addressed are: 1) Does considerable differentiation occur on drought-responding EST-SSR loci between upland and lowland rice ecotypes? 2) Is the differentiation between upland and lowland rice related to drought-resistance? 3) How do the upland- and lowland-differential alleles of the selective loci respond to drought stress? Answering these questions will facilitate our understanding of the evolution of drought-resistance in rice and give cues in rice breeding for drought-resistance.

## Materials and Methods

### Plant materials

A total of 377 rice landraces from Yunnan, Guizhou, Guangxi, Jiangsu, and Hebei province in China were used to study the differentiation between upland and lowland rice ecotypes (Table 1). Based on the information provided by the National Core Germplasm Database (http://crop.agridata.cn/A010110.asp), the landraces of upland rice from the five provinces were accounted for ∼80% of the total upland rice germplasm in China. The predefined subspecies and ecotypes of rice landraces were provided by the institutes that collected them. Thus, crop materials in this experiment can be divided into four groups: *jap*-upland, *jap*-lowland, *ind*-upland, and *ind*-lowland. Additionally, 22 common wild rice strains were also used as reference while studying the differentiation among these two rice ecotypes. Twenty-two drought-resistant traits of 56 *japonica* and 49 *indica* materials were further evaluated in the field experiments rice landraces.

**Table 1:** Summary of rice landraces used in the genotyping experiment and their inferred subspecies, ecotypes, and sources.

Groups Regions	*Japonica*		*Indica*	
	Upland	Lowland	Upland	Lowland
Guangxi	15	13	66	33
Yunan	15	6	15	24
Guizhou	42	17	12	9
Jiangsu	14	24	20	11
Hebei	22	19	0	0
Total	108	79	113	77

### Rice DNA extraction and SSR genotyping

Rice genomic DNA was extracted from the 10-day-old seedlings following the common cetyltrimethyl ammonium bromide (CTAB) protocol. Three individual seedlings of a landrace were mixed together to include the genetic variations within a material. To focus on the drought-related genetic features, 47 polymorphic drought-responding EST-SSRs (SSRs locating in drought-responding ESTs) across 12 rice chromosomes were selected for genotyping (Table S1). The three primers system was applied in the polymerase chain reaction (PCR), containing a pair of EST-SSR primers and a rice-universal M13 sequence. The M13 sequence (5′ to 3′: CACGACGTTGTAAAACGAC) was labeled with fluorescent dyes at the 5′ end. The forward primer of the EST-SSR was also joint with the rice common M13 sequence at the 5′ end. The 20 ul reaction system contains 1×buffer (Mg2+), 2 mM each of dNTP, 50 ng of genomic DNA, 1 U of Taq polymerase, 16 mM of fluorescent dye labeled M13 sequence, 4 mM of the SSR primer liked with M13, and 20 mM of the other SSR primer. The PCR program started with a denaturation period of 4 min at 94°C, followed by 34 cycles of 40 s at 94°C, 30 s at their respective Tm (Table S1), and 40 s at 72°C, and ended after 10 min final extension at 72°C. The PCR products were then analyzed on ABI 3130XL (Applied Biosystems, USA) using ROX500 as the internal standard. The resulting chromatograms were analyzed and scored by Peakscanner ver. 1.0.

### Field drought-resistance evaluation

The field evaluation of the drought-resistant traits was conducted at the Zhuanghang experimental station of Shanghai Academy of Agriculture Science in 2012. Two treatments were designed as control (CK) and drought (DT). Plants in CK were grown in a paddy field following the normal irrigated rice cultivation, while plants in DT were grown in a similar field nearby. The surface level of the DT field was approximately 2 meters higher than the CK field to simulate the upland condition and drain away the water more quickly. The DT field had a drought-resistance screen facility, which equipped a moveable platform capable of stretching out to keep the rainfall away when necessary. The germinated seeds were directly seeded both in the CK and DT on May 30^th^. However, water in the DT field was drained away on July 15^th^ and it was never irrigated until August 25^th^. Individuals of each rice landrace were planted in a plot with 3×4 grids of 20 cm intervals between two seedlings. Consequently, there were 105 plots in one experimental block. Three replicates were included and arranged following the randomized complete block design. The number of stomas per leaf area, the excised leaf water (EWR) loss rate in 4 hours, and the root-shoot ratio were measured from leaves of main tillers in CK 30 days after the beginning of drought treatment. Relative water content (RWC) of leaf samples was measured 16 days after drought both in CK and DT. The content of malonaldehyde (MDA) was measured from fresh leaf tissues 40 days after drought following the protocol of the test kit (Nanjing Jiancheng Bioengineer Institute). The flag leaf length and width were measured at the heading stage both in CK and DT. All morphological traits were measured from three individuals from each replicate. The yield related traits were measured from four individuals of each replicate after harvest. The general drought resistance of a rice landrace was quantified by the drought-resistant index (DRI) [14]. It was calculated as P_d_/P_C_*(P_d_/P_ad_), where P_d_ is the yield production in drought treatment, P_C_ is the yield production in control treatment, and P_ad_ is the average yield production of total materials in drought treatment.

### Missing data and null alleles in data scoring

For genotyping, any null alleles were genotyped twice to avoid the manipulation errors. 337 putative null alleles were detected out of the total 18753 individual-loci combinations (1.8%), which was much lower than that expected by chance at the 5% level. The putative null alleles were treated as missing data in the further analyses.

### Population structure and genetic diversity analysis

The model-based program STRUCTURE ver. 2.3.3 was used to infer population structure using the admixture model with a burn-in length of 50,000 and a run length of 50,000 for Markov Chain Monte Carlo iterations. Ten simulations were run for each K from K = 1 to K = 8 and the results were generated together using software CLUMPP_Windows.1.1.2. The Evanno's K was applied to determine the inferred K value [15]. The Principal Coordinate Analysis (PCoA) implemented in GenAlex ver. 6.43 was conducted to investigate the genetic distances between rice landraces and wild rice using the total 47 SSR loci. Genetic diversity was quantified by four estimators as gene diversity, number of alleles, heterozygosity, and polymorphism information content (PIC) using the software PowerMarker ver. 3.25 based on the total 47 EST-SSR loci. The standard deviations were calculated from 100 times bootstrap.

### Neutrality tests for EST-SSR loci

Most of the genic markers used in this study were neutral among ecotypes as very low *Fst*s were recorded among rice ecotypes, similar with that using putatively neutral genomic SSRs [11]. Three *Fst*-based outlier tests were included to detect signs of selection among these genic SSRs respectively in the *indica* and *japonica* subspecies as indicated by the population structure. First, LOSITAN was used to detect loci under selection at the 95% confidence level [16]. An initial run with 100,000 simulations was conducted and followed by computing the distribution of neutral *Fst* using the putatively neutral loci derived from the initial run with 500,000 simulations. Second, the hierarchical-Bayesian method developed by Foll and Gaggiotti (2008) was applied to detect outlier loci using the software BAYESCAN (http://cmpg.unibe.ch/software/bayescan/) on the 95% posterior probabilities [17]. The parameters were set to 10 pilot runs of 5,000 iterations and additional burn-in of 50,000 iterations. Third, the software DETSEL 1.0 was applied to do pairwise comparisons to identify the outliers. For DETSEL, the coalescent simulation was conducted with the following parameters: mutation rate (infinite allele model) 0.005, 0.001, and 0.0001; ancestral population size *Ne* = 500, 1,000, and 10,000; population size before the split *N_0_* = 100; time since an assumed bottleneck *T_0_* = 50, 100, and 1,000 generations; and time since divergence *t* = 100 generations. Loci falling outside the specified “probability region (95% levels)” were considered as outliers [18]. Based on these outlier tests, two levels of loci sets could be defined from the total loci set as: (1) the neutral loci, which were not detected in any of the three tests, and (2) the decisive selective loci, which were detected at least twice by the three outlier tests.

### Genetic differentiation among upland and lowland rice

The general level of genetic differentiation among different groups was quantified by pairewise *Fst* calculated in Arlequin ver. 3.1 using total loci set with 1,000 permutations. To estimate the level of differentiation on selective loci, *Fst* was calculated from the decisive selective loci set among upland and lowland ecotypes in *japonica* and *indica*, respectively.

*Japonica* landraces were from 5 provinces and *indica* landrace were from 4 provinces. Thus, there were 10 *japonica* and 8 *indica* populations included in this study. To explore the influence of the genetic differentiation on population structures, genetic distance (GD) matrices were calculated by GenAlex ver. 6.43 with 999 permutations using the corresponding neutral or decisive selective loci. The genetic distance matrix obtained form the calculation was used for the cluster analysis according to the un-weighted pair-group method with arithmetic averages (UPGMA) *via* the software NTSYS ver. 2.10e.

### Testing whether the upland-lowland rice differentiation was related to drought-resistance

According to previous studies, the comparison of quantitative genetic divergence (*Qst*) and neutral genetic divergence (*Fst*) can be used to detect adaptive evolution. If the *Qst* is significantly higher than the *Fst* calculated by the neutral loci, it indicates that the directional selection drives phenotypic divergence and results in ecological adaptation [19]. In this study, the *Qst* of each trait was calculated as: *Qst* = V_pop_/(V_pop_+2V_ind_), in which V_pop_ was the variance among-population and V_ind_ was the variance within-population. Although the SSRs located in the ESTs, the *Fst* generated among upland and lowland rice was as low as genomic SSRs [11]. This suggests most of these markers were neutral among the two ecotypes. The 95% confidence interval of *Qst* was calculated using R program with 1000 bootstraps. The *Fst* derived from neutral loci and its standard errors were calculated by Arlequin ver. 3.1 with 1000 permutations. Any significant differences between the *Qst* and *Fst* at *p* = 0.05 level was determined when |*Qst−Fst*|>2SQRT (SE_Qst_^2^+SE_Fst_^2^).

To test whether the genetic differentiation between upland and lowland rice was resulting from divergent selections on drought-resistance, Mantel tests were conducted between the individual based GD matrix using the neutral or decisive selective loci and matrix constructed from differentiated morphological traits. The differentiated traits were defined as the traits having significant differences between upland and lowland rice by independent *t*-test using SPSS ver. 15.0. The individual based GD matrix was constructed by GenAlex ver. 6.43. The dissimilarity matrices of differentiated traits were constructed by Hierarchical Cluster Analysis on SPSS ver. 15.0.

### Test the expression of ecotype-specific alleles of the selective loci

The decisive selective loci may play a role during upland rice adapting to water-limited upland conditions. To test this hypothesis, the expression levels of upland- and lowland-preferable alleles of the decisive selective loci (E647 and E1899 in *japonica*, E647 and E1177 in *indica*) were measured. Here, upland- or lowland-preferable allele was defined as a predominant allele in upland or lowland rice whose frequency was 20% higher than that in lowland or upland ecotype. The allele frequency was calculated as the number of counts of an allele/the total number of counts of all alleles in a given group *via* the software GenAlex ver. 6.43. Four landraces of each ecotype conferring the ecotype-preferable alleles were included. Three-week-old seedlings in the growth chamber were treated with 20% polyethylene glycol (PEG) 6000 to cause osmotic stress (DT). Some materials were kept in nutrition solutions as controls (CK). When most of the seedlings showed signs of leaf rolling 5 hours after treatment, leaves of three seedlings in DT and CK were collected and kept in liquid nitrogen until RNA extraction. The RNA extraction was followed by the kits of TRNzol A^+^ (TianGen Biotech Co. Ltd.). The level of gene expressions were quantified by RealTime-PCR using SYBR^R^ premix Ex Taq (Takara Bio. Inc.). *Actin* was used for reference. The expression change value was calculated as: the expression level in DT over that in CK. In addition, to test whether the ecotype-preferable alleles of the selective loci had any impacts on rice drought-resistance, the drought-resistant traits were further compared among upland and lowland rice containing differently expressed ecotype-preferable alleles (E647 and E1899 in *japonica*, and E1177 in *indica*).

## Results

### Population structure and general genetic diversity

**Figure 1:**
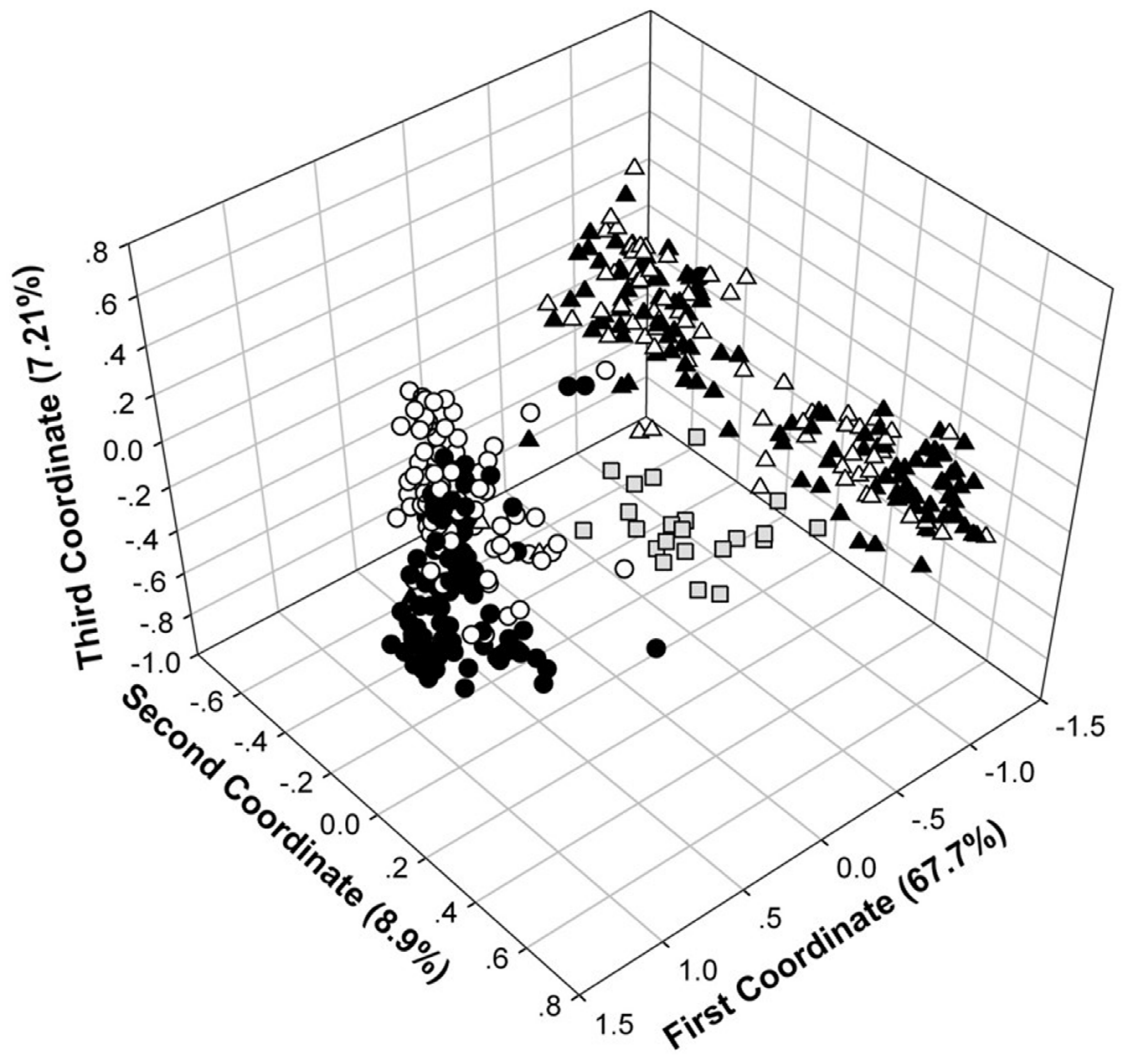
Population structure and differentiation investigated by Principal Coordinate Analysis. White color indicated lowland rice; dark color indicated upland rice; grey color and square indicated wild rice. Circles and triangles indicated *japonica* and *indica* subspecies, respectively.

A total of 477 alleles were detected in the 47 drought responding EST-SSR loci with number of alleles per locus ranging from 4–25. When running the STRUCTURE simulation using the total materials, there was a sharp peak in Evanno's ΔK at K = 2 (ΔK = 3718.5) which divided all materials into *japonica* and *indica* groups (Figure S1a). In *japonica*, the sharp peak occurs when K = 2 (ΔK = 7.6), which divided landraces into two groups as upland and lowland rice occupied the majority in each (Figure S1b). In *indica*, the sharp peak was also occurs when K = 2 (ΔK = 2760.1), which separated rice landraces of Guangxi from other regions (Figure S1c). However, there was a lower peak when K = 4 (ΔK = 78.0), in which upland rice (white) was distinguishable from lowland rice (thin gray) in Guangxi (Figure S1d). Similar to the results of STRUCTURE, PCoA indicated that *japonica* and *indica* landraces were separated along the first coordinate (x-axis) while common wild rice located at the center (Figure 1), suggesting that upland-lowland rice differentiation should be separately analyzed in *japonica* and *indica* subspecies. Differentiation between upland and lowland ecotypes was more apparent in *japonica* than that in *indica* (Figure 1). The level of genetic diversity was similar between upland and lowland rice ecotypes, while subspecies (*japonica vs. indica*) and species (*O. sativa vs. O. rufipogon*) showed considerable differences (Table 2).

**Table 2:** Four genetic diversity estimators (mean ± standard deviations) analyzed in five groups using total loci set by PowerMarker ver. 3.25.

Group	Sample size	Allele No.	Gene Diversity	Heterozygosity	PIC
*Jap*-upland	108	6.88±0.59 b	0.435±0.030 a	0.036±0.008 b	0.399±0.032 a
*Jap*-lowland	79	6.49±0.46 a	0.428±0.040 a	0.024±0.004 a	0.402±0.033 a
*Ind*-upland	113	6.94±0.43 b	0.489±0.033 b	0.033±0.006 b	0.440±0.028 b
*Ind*-lowland	77	6.46±0.38 a	0.511±0.028 c	0.033±0.006 b	0.466±0.026 c
*O. rufipogon*	22	7.32±0.46 c	0.676±0.028d	0.336±0.023 c	0.636±0.025d
Overall	399	10.12±0.78	0.642±0.022	0.051±0.006	0.586±0.024

The different letters behind the values indicated significant differences (p<0.05) among groups by one-way ANOVA.

### Selective loci detected by neutrality tests

Among the 47 drought-induced EST-SSR loci, 5, 9, and 5 loci were detected to be selective by Lositan, BayeScan, and Detsel, respectively, in *japonica* subspecies. Five loci were detected to be the decisive selective loci (Table 3). In the *indica* subspecies, 2, 2 and 7 loci were detected to be selective by Lositan, BayeScan, and Detsel, respectively. Three loci were considered as the decisive selective loci in *indica* (Table 3).

**Table 3:** Outlier loci detected under selection among upland and lowland ecotypes at 95% confidence by three outlier tests.

Method/Software	Code of locus	
	*Japonica* group	*Indica* group
Lositan	***E359, E647, E1238, E1899, E3735***	***E1177***, ***E4208***
BayeScan	E214, E385, E674, E986, E1161, E1177, E1188, ***E1238***, E1615	***E647***, E1188
Detsel	***E359***, ***E647***, ***E1899***, E1941, ***E3735***,	E399, ***E647***, ***E1177***, E1350, E1719, ***E4208***, E4632

Loci in **bold** and *italic* indicated they were detected at least twice.

### Genetic differentiation among upland and lowland rice

**Figure 2:**
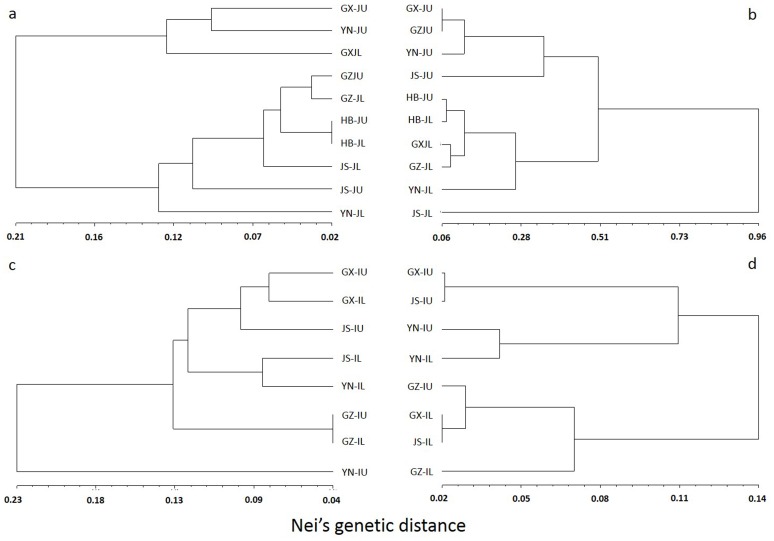
Regional populations cluster analysis using UPGMA method. a) *japonica* materials using neutral loci, b) *japonica* materials using decisive selective loci, c) *indica* materials using neutral loci, d) *indica* materials using decisive selective loci. GX, Guangxi; GZ, Guizhou; HB, Hebei; JS, Jiangsu; YN, Yunnan. U = upland; L = lowland.

The values of *Fst* were as low as 0.0714 in *japonica* or 0.0302 in *indica* types of upland and lowland rice. The value was much lower than that between *japonica* and *indica* varieties (Table 4). This result was similar to a previous study in which *Fst* among different ecotype was recorded as 0.068 in *japonica* using putative neutral SSR markers [11]. However, the values of *Fst* between upland and lowland rice became much higher in *japonica* (0.285) and *indica* (0.127) when using their respective decisive selective loci. In the UPGMA clusters, upland and lowland rice populations were grouped separately when using the decisive selective loci, while populations of landraces from the same regions were clustered together when the neutral loci were used (Figure 2). These results suggest differentiation occurred between upland and lowland rice generally on the selective loci.

**Table 4:** Pairewise *Fst* among five experimental groups using total loci set.

Group	*Jap*-upland	*Jap*-lowland	*Ind*-upland	*Ind*-lowland	*O. rufipogon*
*Jap*-upland	0.0000	+	+	+	+
*Jap*-lowland	0.0714	0.0000	+	+	+
*Ind*-upland	0.4426	0.4649	0.0000	+	+
*Ind*-lowland	0.4255	0.4475	0.0302	0.0000	+
*O. rufipogon*	0.2683	0.2798	0.2405	0.2207	0.0000

### Divergent selection on drought-resistant traits

Drought resistant traits were evaluated in the field experiment. Significant differences were detected on 11 traits between upland and lowland ecotypes in *japonica*, while significant differences were detected on only 2 traits in *indica* (Table 5). The comparison between *Qst* with neutral *Fst* was used to test any divergent selection on the drought-resistant traits. The values of the *Qst* ranged from 0.009∼0.171 in *japonica* and from 0.009∼0.072 in *indica*. The *Qst*s of the most differentiated traits were significantly higher than the neutral *Fsts* in *japonica* (0.0898±0.0024) and *indica* (0.0363±0.0017) materials (Table 5). This result suggested that some of the drought-resistant traits were under divergent selection during domestication.

**Table 5:** 22 drought-resistant traits measured in control treatment (CK) and drought treatment (DT) and their *Qst* calculated in *Jap*-upland/lowland and *Ind*-upland/lowland pairs (mean ± standard error).

Traits	Jap-upland	Jap-lowland	J-*Qst*	Ind-upland	Ind-lowland	I-*Qst*
RWC (CK)	0.936±0.005*	0.910±0.010*	0.052±0.0012	0.898±0.010	0.919±0.006	0.034±0.0007
RWC (DT)	0.871±0.006	0.853±0.011	0.032±0.0010	0.830±0.009	0.843±0.009	0.021±0.0007
No. of stomas per area	13.9±0.3*	15.1±0.5*	0.048±0.0012	14.8±0.3	14.7±0.4	0.011±0.0005
EWR	0.199±0.011	0.194±0.009	0.008±0.0004	0.219±0.011	0.219±0.011	0.010±0.0004
Root-shoot ratio	0.215±0.006*	0.259±0.014*	0.091±0.0016	0.225±0.009	0.239±0.014	0.017±0.0007
MDA (CK)	0.97±0.02	0.91±0.02	0.016±0.0006	0.90±0.02*	0.97±0.02*	0.072±0.0016*
MDA (DT)	1.28±0.02	1.30±0.02	0.043±0.0010	1.27±0.02	1.26±0.02	0.010±0.0004
Flag leaf length (CK)	35.9±1.1	33.2±1.1	0.033±0.0010	36.0±1.5	38.0±1.6	0.020±0.0008
Flag leaf length (DT)	32.1±1.2*	24.5±1.1*	0.171±0.0019*	32.1±1.6	28.5±2.2	0.032±0.0010
Flag leaf width (CK)	1.62±0.05*	1.33±0.05*	0.131±0.0021*	1.45±0.05	1.51±0.05	0.016±0.0006
Flag leaf width (DT)	1.70±0.06*	1.31±0.07*	0.151±0.0015*	1.33±0.07	1.35±0.06	0.009±0.0004
No. of panicles (CK)	6.65±0.32*	9.70±0.73*	0.141±0.0021*	10.54±0.53	10.65±0.53	0.009±0.0004
No. of panicles (DT)	6.01±0.35	7.05±0.40	0.043±0.0011	11.12±0.67	10.24±0.85	0.018±0.0007
100-grain weight (CK)	2.36±0.06	2.50±0.07	0.030±0.0010	2.39±0.04	2.30±0.06	0.026±0.0009
100-grain weight (DT)	2.43±0.06	2.54±0.07	0.022±0.0008	2.25±0.04*	2.07±0.07*	0.066±0.0015*
No. of seeds (CK)	551.6±32.5*	728.2±48.2*	0.096±0.0017*	903.8±39.6	1079.7±70.2	0.051±0.0013*
No. of seeds (DT)	480.0±29.3	445.7±29.5	0.014±0.0006	755.8±45.0	840.8±54.8	0.026±0.0010
Seed-set rate (CK)	0.791±0.016*	0.851±0.012*	0.086±0.0015	0.827±0.009	0.839±0.015	0.020±0.0008
Seed-set rate (DT)	0.822±0.009	0.835±0.020	0.018±0.0009	0.838±0.009	0.821±0.016	0.019±0.0006
Yield (CK)	13.13±0.88*	18.17±1.26*	0.108±0.0018*	21.51±0.87	24.56±1.44	0.036±0.0010
Yield (DT)	11.25±0.53	11.21±0.80	0.011±0.0005	16.84±1.02	17.35±1.23	0.012±0.0006
DI	0.89±0.13*	0.57±0.07*	0.042±0.0009	1.02±0.11	0.93±0.12	0.017±0.0007

RWC: leaf relative water content, EWR: excised leaf water loss rate, MDA: malonaldehyde, DI: drought index.

The values in **bold** and with “*” indicated significant differences between upland and lowland ecotypes or significant difference between *Qst*s and the neutral *Fst*.

### The genetic differentiation associated with morphological differentiation on drought-resistant traits between upland and lowland rice

**Figure 3:**
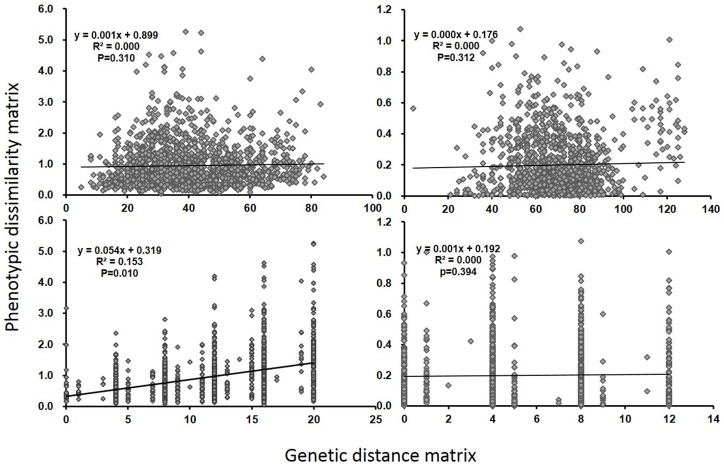
Correlations between matrices of genetic distance from different loci sets and the dissimilarity matrices from selective morphological traits. a) neutral loci in *japonica*; b) decisive selective loci in *japonica*; c) neutral loci in *indica*; d) decisive selective loci in in *indica*.

Given that some drought-resistant traits were differentially selected in upland and lowland rice, their genetic differentiation may be associated with the evolution of drought-resistance. To test this, Mantel test was conducted between the individual-based genetic distance (DG) matrix and dissimilarity matrix constructed from morphological traits. In *japonica*, the dissimilarity matrix constructed from the differentiated traits was significantly correlated with the GD matrix using decisive selective loci (Figure 3b), while it was not correlated with the neutral GD matrix (Figure 3a). These results suggested a strong association between the differentiations on the drought-resistance and on the selective loci in *japonica*. However, the morphological dissimilarity matrix was neither significantly correlated with GD matrix from the selective loci nor with that from the neutral loci in *indica* (Figure 3c, 3d).

### Expression of ecotype-preferable alleles and their impacts on drought-resistant traits

**Figure 4:**
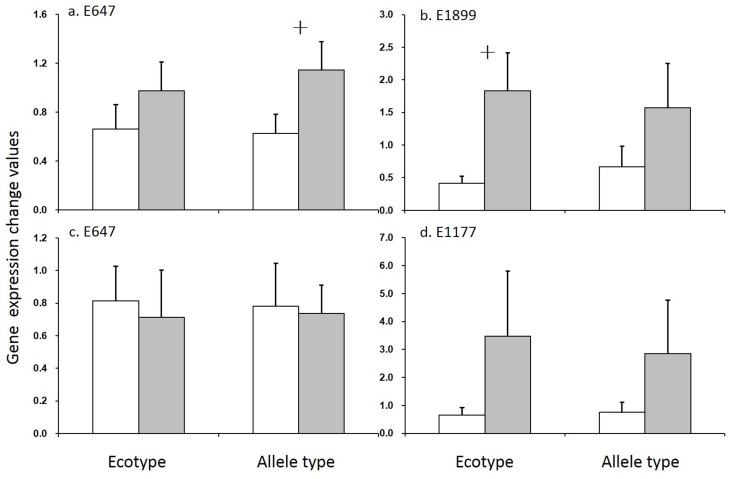
The expression change values (mean ± SE) between different ecotypes or alleles. The expression change values of E647 (a) and E1899 (b) between ecotypes or alleles in *japonica* and these of E647 (c) and E1177 (d) between ecotypes or alleles in *indica*. The expression change values were calculated as: gene expression in PEG/that in water. The white bar indicates upland rice or upland-specific allele, while the black bar indicates lowland rice or lowland-specific allele. “+” indicates differences at the significant level of p<0.10.

In *japonica* subspecies, the allele-6 of locus E647 was upland-preferable (taking account for 87.9% in upland rice but only 50.6% in lowland rice), while allele-2 was lowland-preferable (taking account for 40.4% in lowland rice but 0 in upland rice). These two alleles expressed similarly in normal conditions (CK). However, the expression of allele-6 down-regulated largely (>50%) while the expression of allele-2 remained at the same level in DT. Thus, the expression change values of the allele-2 (1.146±0.232) were marginally higher than the allele-6 (0.625±0.158, p<0.10) (Figure 4a). The allele-4 of E1899 was upland-preferable (taking account for 71.0% in upland rice but only 16.7% in lowland rice) and the allele-3 was lowland-preferable (taking account for 79.5% in lowland rice but 23.8% in upland rice). This EST was up-regulated in lowland rice ecotype while its expression was down-regulated in upland rice under DT. Thus, the expression change value of the allele-3 in lowland rice (1.832±0.583) was marginally higher than the allele-4 in upland rice (0.413±0.111, p<0.10) (Figure 4b).

In *indica* subspecies, the allele-6 of E647 was upland-preferable (taking account for 71.4% in upland rice but 50.7% in lowland rice), while the allele-7 was lowland-preferable (taking account for 49.3% in lowland rice but only 23.7% in upland rice). The allele-8 of E1177 was upland-preferable (taking account for 44.1% in upland rice but only 17.3% in lowland), while the allele-9 lowland-preferable (accounted for 74.7% in lowland rice but only 44.5% in upland). The expression change values of E647 were similar among ecotypes or allelic types (Figure 4c), while that of E1177 was much higher in lowland ecotype or lowland-preferable alleles than in upland rice or upland-preferable (Figure 4d).

As these ESTs differently expressed between allelic types or ecotypes, the drought-resistant traits were then compared among rice ecotypes conferring their ecotype-preferable alleles. As expected, many significant differences not previously detected among total upland and lowland rice were now detected (Table S2). For example, the upland rice conferring the allele-8 and lowland rice conferring allele-9 of E1177 exhibited significant difference on the root-shoot ratio in *indica*. This was accordant to the annotated function of gene *Os06g0633300* (Table 6). Other genes containing the selective EST-SSRs were also considered to be related with resistance to abiotic stress given their annotations (Table 6), suggesting that these selective loci might play roles in rice drought-resistance.

**Table 6:** Gene symbol, gene ID, and the annotated functions of the decisive selective loci detected in this study.

Locus	Gene symbol	Gene ID	Names	Predicted function
E647	Os01g0607400	4324222	hypothetical protein	Similar to STYLOSA protein
E359	Os06g0702600	4341978	hypothetical protein	Similar to Auxin response factor 7a
E1899	Os12g0563600	4352535	hypothetical protein	Protein of unknown function, DUF538 family protein
E3735	Os07g0260000	4342870	hypothetical protein	Protein prenyltransferase domain containing protein
E1238	Os10g0554200	4349339	hypothetical protein	TGF-beta receptor, type I/II extracellular region family protein
E1177	Os06g0633300	4341588	hypothetical protein	Phytosulfokines 1 precursor [Contains: Phyto sulfokine-alpha (PSK- alpha) (Phytosulfokine-a); Phytosulfokine-beta (PSK-beta) (Phytosulfokine-b)]
E4208	Os07g0546500	4343527	hypothetical protein	Conserved hypothetical protein

## Discussion

### Considerable level of upland-lowland rice differentiation on selective loci

As in previously studies, obvious differentiation was reported on drought-resistant morphological traits between upland and lowland rice [9]. However, the general level of genetic differentiation between upland and lowland rice was very low in our study, similar to the results in previous studies [10]–[11]. However, *Fst* calculated based on the outlier loci was considerably high between upland and lowland rice ecotypes, matching the level of *Fst* between *O. sativa* and *O. rufipogon* (0.252) in our study. These results suggested that adaptive divergence among upland and lowland rice occurred on these selective loci. Strong selections on the adaptive loci always affected population structures [20]–[21]. For example, wheat grown in different ecosystems was clustered separately based on the drought-responding gene TaSnRK2.7 [21]. Similar results were found in this study as revealed by the UPGMA cluster, providing evidences that these selective EST-SSRs received uniform selections among regions. It is noteworthy that morphological differentiation between upland and lowland ecotypes was also greater in *japonica* than in *indica*, consistent with the genetic data in these two subspecies.

### Upland-lowland rice differentiation was driven by divergent selection on drought-resistance

Theoretically, drought-resistance alleles are more strongly selected in upland rice than that in lowland rice, due to its adaption to the water-limited environment. In this study, the general drought-resistance gained from field experiments was much higher in upland rice than that in lowland rice. The higher *Qst* than neutral *Fst* suggests some drought-resistant traits are under the divergent selections, leading genetic differentiation on the outlier loci between upland and lowland rice. The genetic differentiation between upland and lowland rice is likely associated with drought-resistance based on the Mantel tests between the genetic distance and morphological dissimilarity, especially in *japonica*. The formation of upland- or lowland-preferable alleles always result from divergent selections [22]. Different expression of the ecotype-preferable alleles under their favorable conditions could be considered as the signature of natural selection [23]. In this study, we found different expression patterns among upland- and lowland-preferable alleles encountering osmotic stress, adding further evidence of the adaptive divergence between upland and lowland rice. Based on these results, we conclude that genetic differentiation between upland and lowland rice is likely driven by divergent selection on the drought-resistance.

### Candidate genes for drought-resistance gene from the selective loci

Recently studies disclosed that it was possible to indentify potential functional genes *via* studying adaptive divergence in natural populations [24], crops [5], and even in rice [25]. In this study, we found potential candidate genes that may play roles in rice drought-resistance. For example, *Os06g0633300* (E1177) encodes the rice phytosulfokine 1 precursor (*OsPSK1*). Its over-expression can promote rice cell division [26] and root development [27]. Interestingly, upland and lowland rice conferring different ecotype-referable alleles of E1177 exhibited significant differences on root-shoot ratio, a key character of drought-avoidance. Besides, genes with similar annotations as *Os01g0607400* (E647) and *Os12g0563600* (E1899) were also reported to be associated with plant stress responses by previous studies in other plant species [28]–[29]. These results provide strong evidences that these candidate genes should have played some roles in rice drought-resistance. However, their functions in rice need further investigation. In a word, we can explore more drought-resistant genes by studying the genetic divergence among the upland and lowland rice.

### Conclusions

Crops adapted to different agro-ecosystems always promote the variation of agricultural important genes. In this study, several outlier loci were detected between upland and low rice ecotypes. A considerable degree of genetic differentiation between upland and lowland rice was detected both at the DNA and gene expression level on these outlier loci. Results from this study reveals that the genetic differentiation among the two rice ecotypes is most likely driven by divergent selection on drought-resistant traits. The findings of this study not only help us to understand the underlying molecular basis of adaptive divergence, but also provide valuable implications for rice domestication and breeding, especially on the drought-resistance in rice.

## Supporting Information

Figure S1 **Population structures inferred by STRUCTURE.** a) *Japonica* and *indica* subspecies were separated when K = 2. b) Inferred population structures in *japonica* subspecies when K = 2. c) Inferred population structures in *indica* subspecies when K = 2. d) Inferred population structures in *indica* subspecies when K = 4, in which some upland rice and lowland rice were separated.
(JPG)

Table S1 **Basic information of the used 47 drought-induced EST-SSR loci.**
(DOC)

Table S2 **22 drought-resistant traits measured in upland and lowland rice materials containing their ecotype-preferable alleles (indicated in parentheses) in control treatment (CK) and drought treatment (DT) (mean ± standard error).** The values in bold and with “*” indicated significant differences between upland and lowland ecotypes.
(DOC)

Table S3 **Original data for SSR scoring and field evaluated traits (xls).**
(XLS)
