# Supplementary figures and images for: Genetic Differentiation Revealed by Selective Loci of Drought-Responding EST-SSRs between Upland and Lowland Rice in China
Source: PLoS One. 2014 Oct 6;9(10):e106352. doi: 10.1371/journal.pone.0106352 (PMC4186790; doi:10.1371/journal.pone.0106352)

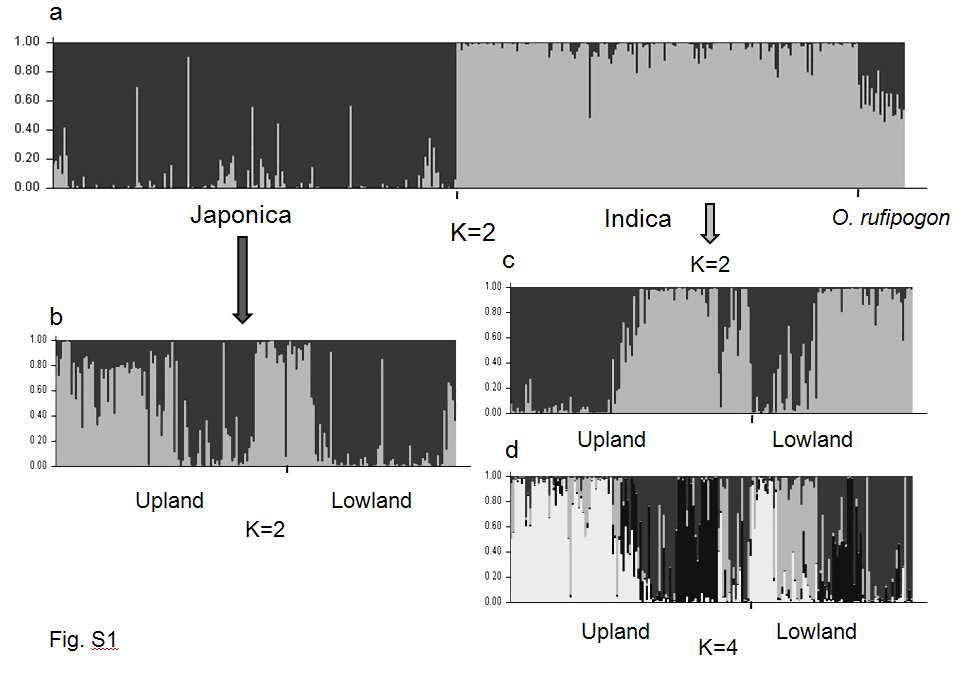

Supplement: Figure S1 — Population structures inferred by STRUCTURE. a) Japonica and indica subspecies were separated when K = 2. b) Inferred population structures in japonica subspecies when K = 2. c) Inferred population structures in indica subspecies when K = 2. d) Inferred population structures in indica subspecies when K = 4, in which some upland rice and lowland rice were separated. (JPG) [file pone.0106352.s001.jpg]
