# Supplementary material for: Genetic Differentiation Revealed by Selective Loci of Drought-Responding EST-SSRs between Upland and Lowland Rice in China
Source: PLoS One. 2014 Oct 6;9(10):e106352. doi: 10.1371/journal.pone.0106352 (PMC4186790; doi:10.1371/journal.pone.0106352)
Supplement: Table S1 — Basic information of the used 47 drought-induced EST-SSR loci. (DOC) [file pone.0106352.s002.doc]

**Table S1.** Basic information of the used 47 drought-induced EST-SSR loci.

| Name | Origin ID* | Motif | Chr. | Primer sequences (5’→3’) | Tm (℃) |
| --- | --- | --- | --- | --- | --- |
| E1057 | TC108346 | AT | 1 | F: GCAACCTACTTCCACCCTCA; R: TCAGATTTGTATTTCATTCTTGAGC | 55 |
| E2011 | TC107900 | AAAT | 1 | F: TGAGCACACACACGCTCATA; R: CTGAAACCCCGATATAGGCA | 55 |
| E647 | TC105859 | AGC | 1 | F: GACTCAGCACATGAAAGCCA; R: GTACTCTGGTTTTGCCGCAT | 57.5 |
| E1348 | TC105044 | AAG | 1 | F: TTGGGTGATTTCTTGGAAGC; R: CCTACGAAACTACCGAAGCG | 55 |
| E424 | TC97771 | AAT | 1 | F: AAGCACACACCCACACAAAA; R: ATGGGAGATCCGGCTAGTG | 57.5 |
| E1491 | TC97772 | AAT | 1 | F: AAGCACACACCCACACAAAA; R: GATGGGAGATCCGGCTAGTA | 57.5 |
| E4199 | CA763819 | AATT | 2 | F: GGGTCATGCCGACTAATGAA; R: GTCCTCCAAGCAATACGTCC | 56 |
| E385 | TC97082 | AG | 2 | F: CCATCCACCAACGAAGAAAA; R: CGACGAGGTCGAGTAGAAGG | 57.5 |
| E3480 | BF430705 | ATGC | 2 | F: GACAGGGTGGCAAAGGACTA; R: CGTGCAGAGAAAACAAACCA | 57.5 |
| E1579 | TC97379 | AGC | 2 | F: CTCTTCCCTCTCACCACAGC; R: GGAGAGGGACGCCATCAT | 57.5 |
| E201 | TC106537 | AGG | 3 | F: GCTGATAGCGAGGTGGGTAG; R: CGTACTCGTAGAGCTTGCCC | 57.5 |
| E4208 | CA765821 | ACG | 3 | F: CTAGCTGCTAAACGGCTGCT; R: CCTGCCTTCTCCTACTGCTG | 57.5 |
| E1618 | TC98497 | AGC | 3 | F: TCAGGTGGTCGGTTCCTTAC; R: TTCTCTGGGCACAGGTTCTT | 55 |
| E337 | TC111077 | AGG | 4 | F: ACCGCAATCAACCACAAGAT; R: TTCCTCCTGGAACAACGAGT | 56 |
| E4632 | CA765893 | ACG | 4 | F: GATGGAATGGAATGGAATGG; R: GTGCACACCTCATCCTTGC | 56 |
| E4631 | CA765877 | AAG | 4 | F: GGTTCGTCTTGGCTGATGAT; R: AAGAAAGGGAGCTCAGGGAG | 57.5 |
| E362 | TC111845 | AGG | 4 | F: GTAGGAGGAGGAGGATGCTG; R: ATTCTCCCAGACAAGCATGG | 57.5 |
| E986 | TC105039 | AG | 4 | F: CAAATCTCGCCTCGTTTAGC; R: GATCTTCCCCATGTCGATGT | 57.5 |
| E1941 | TC106301 | AAC | 5 | F: CGCACCTCACCTTCTTTCTC; R: TGCTTGCTTTGCTTTCCTTC | 55 |
| E3676 | CA755756 | AGAGG | 5 | F: ACCACTTCACGTGCCCTATC; R: AGTCCATACACGTCGGTGGT | 56 |
| E399 | TC97256 | AG | 5 | F: GCACATAGTCAGAACACCGC; R: CTCGATCTGGATGGTCCACT | 57.5 |
| E1350 | TC105067 | CCG | 5 | F: CCTACGAAACTACCGAAGCG; R: ACCCTGACCGTGTAGTCGTC | 57.5 |
| E4198 | CA763803 | AGG | 5 | F: GTCTTCTTCTCCTCGGCCTT; R: CTGACAAAAACACGCAAGGA | 56 |
| E2031 | TC108393 | AAAAG | 5 | F: AGACAGAAACACCGTTTGGC; R: AATGCCGTACCCCCTAGGTA | 55 |
| E1256 | TC101072 | AAAAG | 6 | F: CTGAGCTCTGTCGAACTCCC; R: GTTACAGTGTTGGCGCAGAA | 57.5 |
| E359 | TC111795 | AAC | 6 | F: AGCTCAGGTCCTTGTCCAAA; R: CTGATTCGCCAGACTTGACA | 57.5 |
| E1177 | TC97976 | AG | 6 | F: GAAAGAAGAAGCAGCAGCAAA; R: GCAGAGTGCCCTAGCTGTTC | 57.5 |
| E1161 | TC97605 | AC | 6 | F: CGTAGAAACTTGGCTGCTCC; R: ACAACCACACAGGGTTGTCA | 55 |
| E1355 | TC105155 | AAAG | 6 | F: TTCCTCTTTTGGCATCCTTG; R: ACAAACCGGAGAACGCTAAA | 55 |
| E620 | TC104930 | CCG | 7 | F: ACAACAGCCACCAACTCCTC; R: CGTTCAGCTCCTCGTAGTCC | 55 |
| E3735 | CA766676 | AG | 7 | F: TGGCCCTCATGAGACATACA; R: TTAAGCAATCAAAGGGGGTG | 56 |
| E674 | TC107120 | AG | 7 | F: TTCGCTATTTGGGATGGAAG; R: CAGCCAACATGCAAAAAGAA | 57.5 |
| E1639 | TC98837 | ACG | 8 | F: GCTTCTTGGCTGTGTGACTG; R: CAGGGATCGAGCTTGAAGAG | 57.5 |
| E1188 | TC98286 | AG | 9 | F: CCACGTTGATAAGCTCATTGC; R: TGGGCACCGAAAATAAAATC | 55 |
| E823 | TC97891 | AAGGG | 9 | F: CCGTTTAGTTTGCTCCAAGC; R: CGTCCATGGCTATGGCTAAT | 57.5 |
| E1760 | TC101639 | ACG | 9 | F: GCTACAACCACCATGCACAC; R: CATCATCACTGATCGGCAAG | 57.5 |
| E19 | TC100794 | ACC | 10 | F: TGGAAACAAACCTGCCTTTT; R: ACTGAATGATTTGGCAAGGG | 55 |
| E214 | TC107010 | AGG | 10 | F: GAGAAGGACAATCTGGAGCG; R: TAATGGTGGTGTGTCGTCGT | 57.5 |
| E698 | TC107936 | AGC | 10 | F: AACAGTTCCAACAGAACCCG; R: TTATTTGCTGATGCTGCGAC | 57.5 |
| E1238 | TC100157 | AAG | 10 | F: CAGGGGGTACGTCTACAAGG; R: TATACCCGGCGAAATACGTC | 55 |
| E208 | TC106908 | AT | 11 | F: CGCCCAAGCCACTCTACTAC; R: TTGCGACATGGGATTACATT | 57.5 |
| E1949 | TC106454 | AG | 11 | F: ATATCTTCCCTGCAACCGTG; R: AGATGATGGAGTGGACGAGG | 55 |
| E1615 | TC98490 | AAG | 11 | F: CAACCAGTGCTTGAGCTTGA; R: AGTAACAGCATCCATCCATCG | 57.5 |
| E1234 | TC100010 | ACGAG | 11 | F: AACACCCCACCTCACCTCTT; R: CTGCCCTCACTTCCAATCTC | 55 |
| E3534 | BI812776 | AAG | 12 | F: GTACACTCTCCTGGGGAGCA; R: AATTGATGTGTAGCCTCGCC | 56 |
| E1719 | TC100663 | AGC | 12 | F: GCAACTACAGCAGCAACAGC; R: CAACCTTGATTCGATGGACC | 55 |
| E1899 | TC105229 | ATC | 12 | F: CCAAGGTCGATGAGGAGAAG; R: AGTGAGATCTCGTGCTGCTG | 55 |

* The origin ID is collected from the reference below: Rota ML, Kantety RV, Yu JK, Sorrells ME (2005). Nonrandom distribution and frequencies of genomic and EST-derived microsatellite markers in rice, wheat, and barley. *BMC Genomics* **6**: 23.
