# Supplementary material for: Genetic Differentiation Revealed by Selective Loci of Drought-Responding EST-SSRs between Upland and Lowland Rice in China
Source: PLoS One. 2014 Oct 6;9(10):e106352. doi: 10.1371/journal.pone.0106352 (PMC4186790; doi:10.1371/journal.pone.0106352)
Supplement: Table S2 — 22 drought-resistant traits measured in upland and lowland rice materials containing their ecotype-preferable alleles (indicated in parentheses) in control treatment (CK) and drought treatment (DT) (mean ± standard error). The values in bold and with “*” indicated significant differences between upland and lowland ecotypes. (DOC) [file pone.0106352.s003.doc]

**Table S2. 22 drought-resistant traits measured in upland and lowland rice materials containing their ecotype-preferable alleles (indicated in parentheses) in control treatment (CK) and drought treatment (DT) (mean ± standard error). The values in bold and with “*” indicated significant differences between upland and lowland ecotypes.**

|  | E647 in *Japonica* | | E1899 in *Japonica* | | E1177 in *Indica* | |
| --- | --- | --- | --- | --- | --- | --- |
| Traits | Upland (allele 6) | Lowland (allele 2) | Upland (allele 4) | Lowland (allele 3) | Upland (allele 8) | Lowland (allele 9) |
| Sample number (N) | 29 | 12 | 17 | 13 | 14 | 18 |
| RWC (CK) | **0.933±0.004*** | **0.895±0.017*** | 0.926±0.006 | 0.914±0.014 | 0.912±0.009 | 0.915±0.006 |
| RWC (DT) | **0.870±0.006*** | **0.833±0.014*** | 0.868±0.009 | 0.858±0.017 | 0.827±0.010 | 0.846±0.010 |
| No. of stomas per area | **14.1±0.32*** | **16.2±0.68*** | **14.3±0.37*** | **16.3±0.50*** | 13.8±0.52 | 14.8±0.41 |
| EWR | 0.200±0.012 | 0.173±0.011 | 0.192±0.015 | 0.189±0.015 | 0.234±0.016 | 0.212±0.011 |
| Root-shoot ratio | 0.216±0.007 | 0.234±0.018 | **0.203±0.008*** | **0.273±0.023*** | **0.198±0.012*** | **0.248±0.017*** |
| MDA (CK) | 0.967±0.025 | 0.894±0.030 | 0.966±0.037 | 0.895±0.025 | **0.891±0.015*** | **0.980±0.025*** |
| MDA (DT) | 1.280±0.022 | 1.293±0.026 | 1.283±0.025 | 1.311±0.034 | 1.278±0.028 | 1.267±0.021 |
| Flag leaf length (CK) | **35.88±1.14*** | **31.38±1.10*** | 36.94±1.54 | 33.50±1.82 | 37.16±2.39 | 39.15±1.76 |
| Flag leaf length (DT) | **32.59±1.31*** | **22.72±0.95*** | **33.59±1.85*** | **24.90±1.55*** | 30.34±2.18 | 8.83±2.62 |
| Flag leaf width (CK) | **1.63±0.05*** | **1.19±0.04*** | **1.66±0.07*** | **1.28±0.07*** | 1.43±0.08 | 1.54±0.05 |
| Flag leaf width (DT) | **1.72±0.07*** | **1.22±0.06*** | **1.78±0.06*** | **1.23±0.08*** | 1.30±0.11 | 1.37±0.06 |
| No. of panicles (CK) | **6.52±0.33*** | **10.93±1.09*** | **6.40±0.37*** | **9.95±0.62*** | 9.51±0.63 | 10.55±0.56 |
| No. of panicles (DT) | **5.97±0.39*** | **7.55±0.57*** | **5.93±0.49*** | **7.71±0.61*** | 11.26±1.04 | 10.11±0.84 |
| 100-grain weight (CK) | **2.31±0.06*** | **2.59±0.05*** | 2.30±0.08 | 2.49±0.06 | 2.42±0.06 | 2.29±0.38 |
| 100-grain weight (DT) | 2.38±0.06 | 2.58±0.08 | 2.33**±**0.08 | 2.52±0.06 | 2.19±0.07 | 2.06±0.63 |
| No. of seeds (CK) | **539.4±33.2*** | **806.5±80.0*** | **561.1±48.8*** | **732.5±38.8*** | **790.4±66.6*** | **1108.4±74.3*** |
| No. of seeds (DT) | 488.2±31.9 | 453.0±47.9 | 548.2±42.0 | 512.7±36.0 | 773.1±76.5 | 875.8±61.2 |
| Seed-set rate (CK) | **0.789±0.017*** | **0.875±0.011*** | 0.765±0.024 | 0.830±0.018 | 0.836±0.014 | 0.834±0.016 |
| Seed-set rate (DT) | 0.820±0.010 | 0.848±0.025 | 0.816±0.010 | 0.860±0.023 | 0.833±0.011 | 82.9±0.011 |
| Yield (CK) | **12.56±0.84*** | **20.82±1.95*** | **13.16±1.32*** | **18.15±1.03*** | **18.91±1.45*** | **25.26±1.51*** |
| Yield (DT) | 11.23±0.59 | 11.45±1.14 | 12.49±0.78 | 13.02±1.05 | 16.77±1.73 | 17.97±1.37 |
| DI | 0.92±0.14 | 0.53±0.10 | 1.14±0.22 | 0.70±0.09 | 1.14±0.16 | 0.94±0.12 |

RWC: leaf relative water content, EWR: excised leaf water loss rate, MDA: malonaldehyde, DI : drought index.
